# Signatures of a Conical Intersection in Two-Dimensional Spectra of a Red-Absorbing Squaraine Dye

**DOI:** 10.1021/jacs.5c10393

**Published:** 2025-08-26

**Authors:** Vittoria Burigana, Edoardo Buttarazzi, Federico Toffoletti, Elisa Fresch, Francesco Tumbarello, Alessio Petrone, Elisabetta Collini

**Affiliations:** † Department of Chemical Sciences, University of Padova, Via Marzolo 1, Padova I-35131, Italy; ‡ Scuola Superiore Meridionale, Largo San Marcellino 10, Napoli I-80138, Italy; § Department of Chemical Sciences, University of Napoli Federico II, Complesso Universitario di Monte S. Angelo, Via Cintia 21, Napoli I-80126, Italy; ∥ Istituto Nazionale Di Fisica Nucleare, Sezione di Napoli, Complesso Universitario di Monte S. Angelo ed. 6, Via Cintia, Napoli I-80126, Italy

## Abstract

Squaraine dyes are promising for dye-sensitized solar cells (DSSCs) due to their strong absorption in the red and near-infrared regions. However, their ultrafast photophysical behavior remains poorly understood. Using two-dimensional electronic spectroscopy (2DES) and ab initio computations, we reveal signatures of a conical intersection (CI) governing excited-state dynamics in a prototypical squaraine dye. This sub-200 fs decay pathway may hinder squaraine performance as dye-sensitizers. Our results provide experimental evidence of a sloped CI landscape and elucidate the role of at least two vibrational modes strongly coupled to electronic degrees of freedom. The passage through a CI emerges as a key relaxation mechanism, shedding light on the complex photophysics of these dyes.

## Graphical abstract

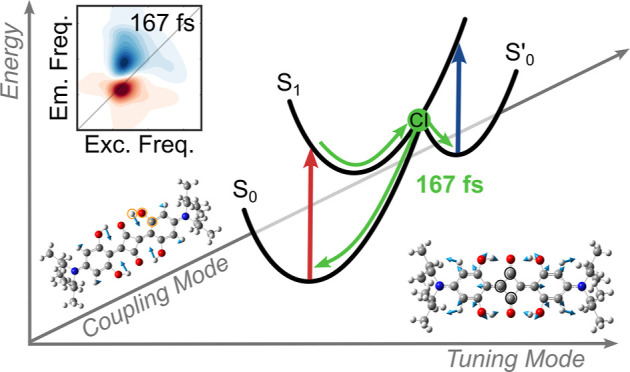

## Introduction

Squaraine dyes have attracted considerable attention in recent years as promising building blocks for organic optoelectronic devices, owing to their intense absorption in the red and near-infrared (NIR) spectral regions, chemical stability, and structural tunability.^1–3^ These dyes, derived from squaric acid, typically feature a donor–acceptor–donor (D–A–D) π-conjugated system composed of a central four-membered ring flanked by electron-donating aromatic substituents. Their strong and narrow absorption profiles, combined with high molar extinction coefficients, make them ideal candidates for photonic applications such as fluorescence imaging, photodynamic therapy, and solar energy conversion.^4^ In the context of dye-sensitized solar cells (DSSCs), squaraines offer several advantages over traditional Ru­(II)-based sensitizers, including cost-effective synthesis, strong absorption in the low-energy region, and a metal-free composition.^5–7^ These features are particularly desirable for improving solar light harvesting under diffuse illumination and enhancing the photovoltage of the device. Nevertheless, despite their promising optical properties and extensive experimental investigation,^2,3^ the understanding of their complex photophysics remains limited, particularly on the femtosecond time scale where critical processes such as internal conversion and charge separation take place. A deeper understanding of nonradiative decay channels is essential, as rapid deactivation pathways can compete with electron injection into the semiconductor in DSSCs. This issue is particularly relevant for red-absorbing dyes, which are more susceptible to undesired relaxation due to their low-energy transitions. To address this challenge, we proposed a combined computational and experimental strategy, by employing both two-dimensional electronic spectroscopy (2DES), to probe the excited-state dynamics of a prototypical symmetric squaraine dye on the femtosecond time scale, and first principle calculations, to provide a molecular interpretation of its ultrafast relaxation.

In this study, we focus on 2,4-bis­[4-(*N*,*N*-diisobutylamino)-2,6-dihydroxyphenyl] squaraine (SQ), whose molecular structure is reported in Figure 1a. This squaraine was selected for its commercial availability and established use in DSSCs that provide an ideal platform for mechanistic investigation. By unraveling the key relaxation pathways in SQ, our work aims to advance the fundamental understanding of squaraine photophysics and inform the rational design of next-generation sensitizers for solar energy conversion.

**Figure 1:**
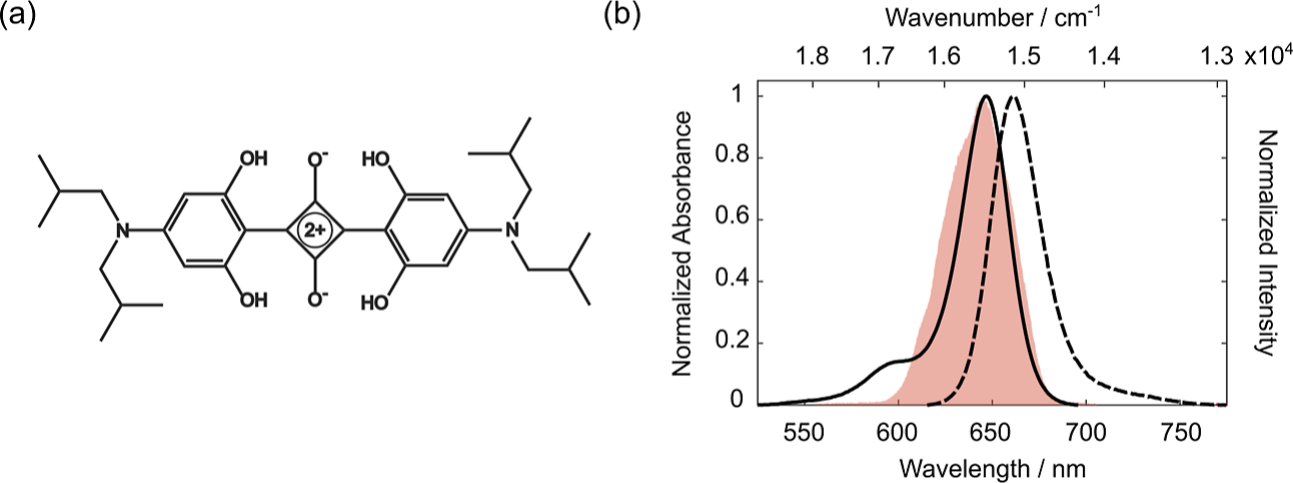
(a) Molecular structure of SQ. (b) Normalized absorption (solid line) and emission (dashed line) spectra of SQ in acetonitrile. The red area represents the laser spectrum profile used in the 2DES experiment.

## Results and Discussion

Preliminary linear optical characterization was performed on SQ dissolved in acetonitrile, and the results are summarized in Figure 1b. The absorption spectrum exhibits a prominent band in the near-infrared region at 647 nm (15,455 cm^–1^), corresponding to the main electronic transition S_1_ ← S_0_, typical of squaraine dyes.^8^ Additionally, a shoulder is observed at 594 nm (16,835 cm^–1^), which, as reported in our previous study, could be attributed to vibronic coupling and conformational distortions of the molecule.^9^ The emission spectrum exhibits a peak at 662 nm and time-resolved fluorescence measurements yielded a fluorescence lifetime of 2.5 ns (see Supporting Information, Section S1). Figure 1b also reports the laser emission profile used for the photoexcitation in the 2DES measurements, which was tuned to overlap with the main electronic transition.

The 2DES measurements were carried out at room temperature using the experimental setup described in ref 10. The temporal resolution, determined via frequency-resolved optical gating (FROG) measurements (see Supporting Information, Figure S2), was estimated to be approximately 10 fs. Further experimental details are provided in the Supporting Information, Section S2. The resulting 2D maps are shown in Figure 2a for selected population times *t*_2_ (additional maps are reported in Supporting Information, Figure S3).

**Figure 2:**
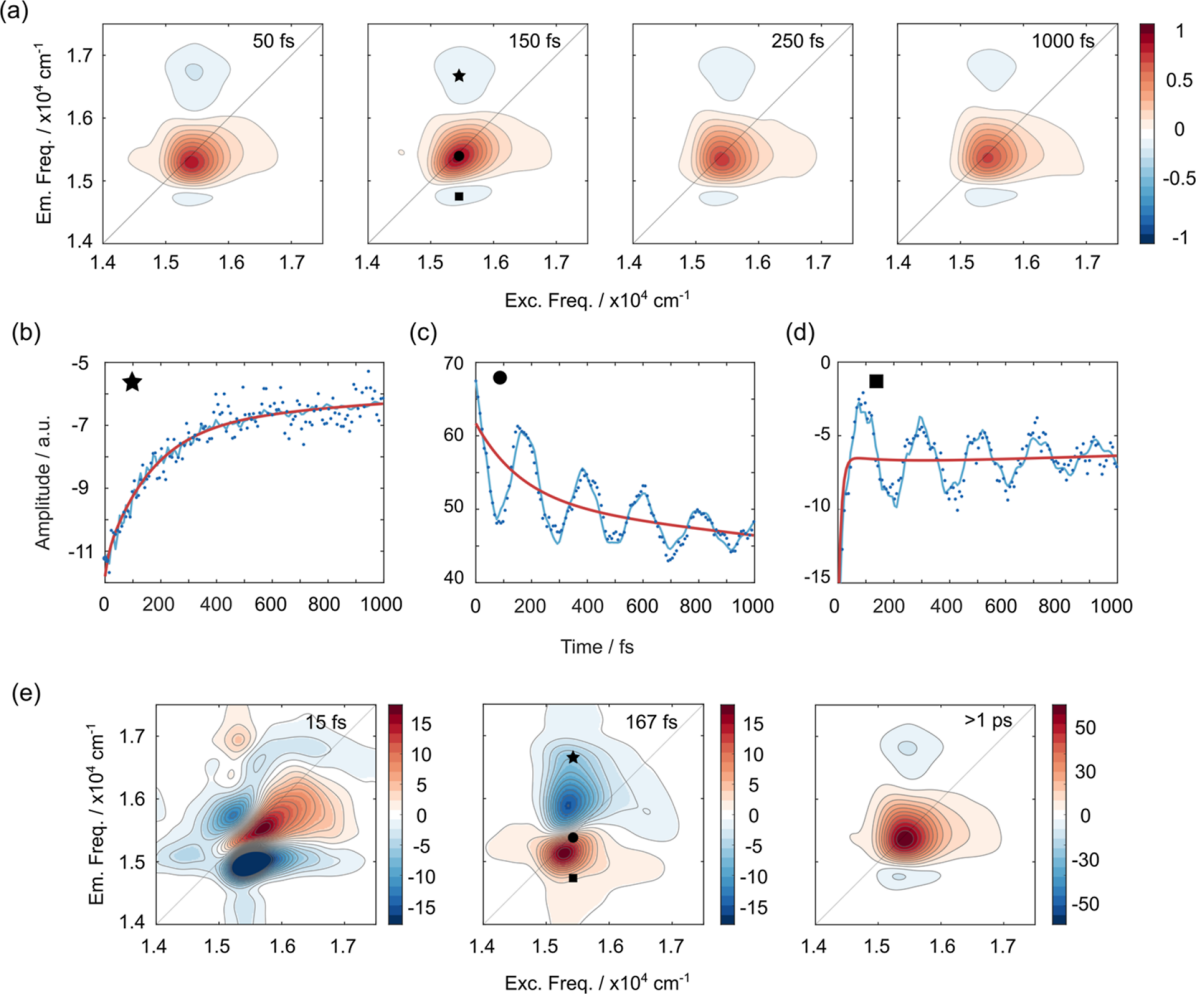
(a) Normalized purely absorptive 2D maps for SQ in acetonitrile recorded at selected values of population times, as indicated in each panel. (b) Signal decay trace extracted at coordinates (15,420, 16,670) cm^–1^ (black star in panel a). (c) Signal decay trace extracted at coordinates (15,420, 15,420) cm^–1^ (black dot). (d) Signal decay trace extracted at coordinates (15,420, 14,760) cm^–1^ (black square). For (b–d), dotted line: experimental data; blue solid line: global fit including both beating and decaying components; red solid line: fit including only decaying components. (e) 2D-DAS of SQ obtained from global fitting of the 2DES data. The corresponding time constants are reported in the panels.

The dominant feature in the 2D maps is a positive diagonal peak centered at 15,420 cm^–1^ (highlighted by the circle marker in Figure 2a), attributed to ground-state bleaching and stimulated emission involving the main electronic transition, S_1_ ← S_0_. Additionally, two off-diagonal negative features appear below and above the diagonal, which can be attributed to photoinduced absorption (PIA) processes. Further insight into the dynamic origin of these PIA signals can be gained by examining the temporal evolution of their amplitude. As shown in Figure 2a, the PIA signal located above the diagonal (pinpointed by the star marker) shows its maximum intensity immediately after photoexcitation, followed by a gradual decay as a function of the population time *t*_2_. This behavior indicates that the signal originates directly from the initially populated first excited state, S_1_, and can thus be assigned to an S_*n*_ ← S_1_ transition. Notably, the decay of this PIA signal closely mirrors the dynamics of the positive diagonal peak, suggesting a correlated depletion of the S_1_ population. This correlation is further illustrated in Figure 2b,c, where the temporal profiles of both signals are compared. In contrast, the PIA signal observed below the diagonal (marked by the square) exhibits distinctly different behavior. Figure 2d shows that the time evolution of the signal amplitude at these coordinates is governed by three distinct time scales. A zoomed-in view of the decay is presented in the Supporting Information, Figure S4. Within the first ∼20 fs, the signal rapidly recovers about half of its amplitude. Between approximately 20 and 400 fs, instead, the amplitude increases (i.e., the signal becomes more negative), suggesting the presence of a sequential process, possibly involving population transfer from the initially excited state to an intermediate state or structural relaxation dynamics. Finally, from 400 fs onward, the signal decays (i.e., the amplitude gradually becomes more positive and approaches zero) with a time constant longer than the time window explored in this study.

To gain deeper insight into the time evolution of the signal, a global multiexponential fitting approach was employed.^11,12^ In this method, the decay of the total complex signal at each point of the 2D map is fitted using a global function expressed as the sum of *N* complex exponentials. These exponentials simultaneously account for both the population decay contributions and the oscillatory components associated with coherent dynamics. This approach enables the simultaneous extraction of frequencies, damping times, and amplitude maps for all fitted components, by considering both the real and imaginary parts of the signal.^11,12^

The nonoscillatory dynamics observed within the investigated time window are best described by three time constants: 15 fs, 167 fs, and ≫1 ps (red fitting trace in Figure 2b–d). The amplitude distributions of these components across the maps are visualized as two-dimensional decay-associated spectra (2D-DAS), reported in Figure 2e. These spectra, when interpreted alongside the 2D maps, help correlate specific dynamical processes with distinct time constants.^13^ In the specific case of PIA signals, characterized by a negative amplitude, a positive peak (red) in a 2D-DAS means that, overall, the signal at those coordinates is becoming more negative. This corresponds to a growth of the population of the state from which the PIA originates, and therefore, we refer to this behavior as a “rising” component.^12^ The opposite is true for negative peaks (blue signals in the 2D-DAS), associated with the decay of the population of the same state. These trends can be easily verified by inspecting the time traces at the coordinates of the main peaks appearing in the 2D-DAS (see Figure 2b–d). The longest time constant describes a decaying component at all the relevant coordinates. It reflects processes occurring on time scales well beyond the experimental window and can be attributed to the restoration of the ground state population in the nanoseconds time scale. This interpretation is consistent with the S_1_ state lifetime, previously measured to be on the order of several nanoseconds via time-resolved fluorescence experiments (Figure S1). The shortest component, with a time constant of 15 fs, corresponds to a decaying contribution across all positions in the maps. This time constant approaches the temporal resolution limit of the experiment and is therefore susceptible to artifacts such as pulse overlap, scattering, and coherent effects. Despite this, the associated 2D-DAS exhibits a characteristic pattern that cannot be entirely attributed to coherent artifacts. Specifically, it shows a positive (red) decaying signal along the diagonal, accompanied by negative (blue) growing signals above and below the diagonal—the latter with notably greater amplitude. This pattern reflects the initial peak broadening (“rounding”) of the main diagonal positive signal and its gradual shift to lower detection frequencies over a time scale of a few tens of fs (see also additional maps in Figure S3). The first effect is typically associated with spectral diffusion^14,15^ and, as expected, this contribution is more pronounced in the rephasing component of the signal^16^ (Figure S5). Furthermore, the pronounced amplitude of the negative feature below the diagonal indicates an ultrafast relaxation from the Franck–Condon region to a more stabilized configuration within the same potential energy surface—an effect that has been previously reported and well-characterized in other red-absorbing dyes.^13,17–20^ Although these relaxation phenomena in organic solvents are generally expected to occur on longer time scales, recent studies have shown that strong anharmonic coupling between high- and low-frequency vibrational modes can significantly modulate^21–25^ or accelerate the process.^26^ This mechanism could also be relevant in the SQ system, as further discussed below. That said, it should be emphasized that the 15 fs component lies very close to the instrument’s resolution limit, and its precise quantification is subject to substantial uncertainty. Therefore, while the assignment to spectral diffusion and rapid intramolecular relaxation within the S_1_ potential energy surface is consistent with multiple pieces of evidence, the exact determination of the associated time constant should be interpreted with caution.

The 167 fs time constant exhibits the most intriguing amplitude distribution across the 2D spectral maps. This component corresponds to a decay of the signal at the diagonal peak (circle marker) and the PIA region above the diagonal (star marker), while simultaneously showing a rise in amplitude in the PIA below the diagonal (highlighted by the black square in Figure 2e). This trend, also anticipated by a preliminary inspection of the time evolution in the 2D maps, suggests a population transfer from the excited singlet state S_1_ to a lower-lying electronic state. The presence of a dark state below S_1_ is unlikely in this quadrupolar system, as suggested by previous studies.^27^ Moreover, the ultrafast character of the 167 fs decay and comparisons with analogous molecular systems suggest that this process could be associated with a conical intersection (CI) between the ground and first excited electronic states, S_0_ and S_1_.^28–31^ CIs are points of degeneracy between adiabatic potential energy surfaces in the multidimensional nuclear coordinate space of polyatomic molecules. These intersections are typically characterized by strong anharmonicity and pronounced nonadiabatic couplings between electronic states, often facilitated by vibrational mode–mode interactions.^32^ The dynamic behavior displayed in Figure 2d closely resembles the amplitude and time scale trends predicted and experimentally observed for CIs in related systems.^31,33^ Previous computational investigations on symmetrical squaraine-based dyes have identified a CI between the S_0_ and S_1_ states, with the associated reaction coordinate involving twisting motions of the lateral donor groups,^34–36^ analogous to the well-established *cis*–*trans* isomerization mechanism. In the case of SQ, the CI could also be linked to a distortion of the central squaric ring, primarily involving carbonyl bond stretching.^37^ This pathway is likely associated with a sloped intersection topography, generally involving nonreactive coordinates^37,38^ and may be correlated with the conformational distortion already predicted for SQ.^9^ Within this framework, the negative peak below the diagonal in the 2D spectrum can be interpreted as a PIA originating from a “hot” ground state (denoted as S_0_^′^) corresponding to a conformational distorted structure populated immediately after passage through the CI (Figure 3).^33,39^ An alternative explanation could involve a broad S_*n*_ ← S_1_ PIA spanning the detection range and overlapping with both ground-state bleach and stimulated emission. In this scenario, the above- and below-diagonal PIA features would arise from the same electronic transition undergoing a dynamic Stokes shift.^13,19,20^ While this interpretation may account for the early time behavior, particularly within the first 15 fs, the subsequent temporal evolution (plotted in Figures 2d and S4), especially on the 167 fs time scale, is not consistent with this mechanism alone. Notably, the signal remains negative throughout the entire time window and becomes increasingly negative with a time constant of 167 fs. This behavior contrasts with the effect of a dynamic Stokes shift, which would cause a blue shift of the broad PIA band, thereby reducing the negative signal amplitude, precisely as observed for the 15 fs component. There are instead two scenarios that can explain a negative signal becoming more negative over time: either the emergence of a new negative contribution, such as the growth of a PIA signal, or the decay of a positive contribution, such as stimulated emission, which reduces its compensating effect and renders the total signal more negative. Both processes—PIA growth and stimulated emission decay from the S_1_ state— are consistent with the mechanism involving a CI crossing. In systems where a CI governs excited-state dynamics, two hallmark photophysical signatures are typically expected: ultrafast nonradiative decay and suppressed fluorescence. While SQ does exhibit a fast nonradiative component, it also displays pronounced fluorescence and a relatively long excited-state lifetime-features that appear inconsistent with a highly efficient CI-mediated decay. A rough estimation based on the amplitudes extracted from the 2D-DAS for the three time components (Figure 2e) indicates that the CI-mediated decay in SQ, corresponding to the 167 fs component, accounts for approximately 20% of the total excited-state relaxation. This partial contribution allows for a substantial radiative pathway, consistent with the observed fluorescence. Similar behavior has been observed in other systems where CI signatures were detected despite strong fluorescence.^40–43^ Fluorescence emission and nonradiative deactivation via CI are inherently competitive processes, and the branching ratio between these two pathways is strongly influenced by the topography of the excited-state potential energy surfaces. This, in turn, depends on several factors, including chromophore conformation and flexibility, the nature and steric hindrance of substituents, environmental interactions, and the hydrogen-bonding network.^37,42,44^ For example, studies on different variants of red fluorescent proteins with varying brightness have shown that increased molecular flexibility and the ability to adopt a pretwisted chromophore conformation facilitate access to CIs, thereby reducing fluorescence efficiency.^42^ It has also been proposed that the accessibility of the CI, and thus the branching ratio between competitive pathways, may strongly depend on the excitation energy and solvation conditions.^44^ In the case of SQ, the inherent rigidity of the molecular backbone and its preferentially planar configuration likely restrict access to the CI, reducing the efficiency of nonradiative decay and favoring fluorescence. Additionally, intramolecular hydrogen bonding involving hydroxyl groups on the phenyl substituents helps stabilize the planar geometry, further reinforcing the emissive state’s stability.^9,45^ Collectively, these structural features make the CI only partially accessible, thereby modulating the nonradiative decay efficiency and supporting the long-lived fluorescence observed in SQ. Further investigations into the role of solvation conditions and the nature of molecular substituents will help clarify the microscopic factors governing the branching ratio between radiative and nonradiative pathways in SQ.

**Figure 3:**
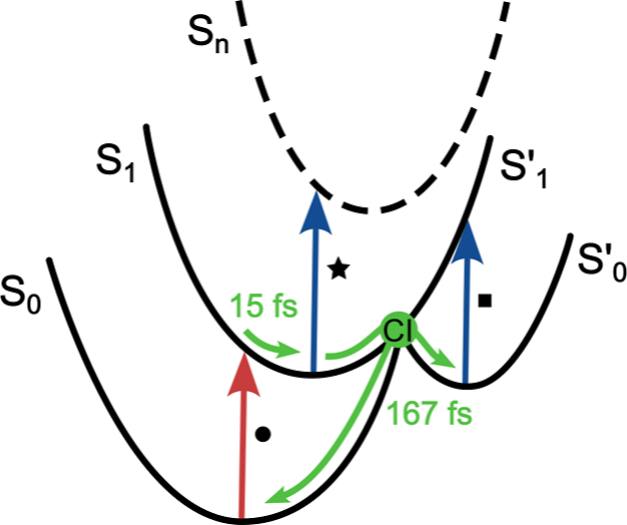
Graphical representation of the conical intersection in SQ. Red (blue) arrows indicate transitions involved in the GSB (PIA) processes; each transition is labeled with the same markers used to identify the corresponding signals in Figure 2. Green arrows schematize wavepacket pathways along the reaction coordinate leading to CI.

The observed behavior of SQ aligns with predictions from the minimal two-state two-mode (2S2M) model, which describes CIs using two coupled electronic states and two vibrational modes: one coupling mode, which enables nonadiabatic interaction, and one tuning mode, which modulates the energy gap between the states.^30,33,39,46^ Given the inherent link between CIs and vibrational dynamics, we performed a detailed analysis of coherent wavepacket evolution, aiming to support the assignment of the observed ultrafast dynamics to a CI-mediated decay pathway. This also allowed us to identify vibrational modes likely involved in the nonadiabatic transition process.^29,30^ In 2D spectra, vibrational coherent wavepackets manifest as signal oscillations as a function of the population time. The beating frequencies that primarily contribute to the overall oscillatory behavior can be preliminarily extracted through an easy Fourier transform (FT) analysis of the oscillatory residuals, obtained by subtracting the nonoscillatory decay components from the signal.^47^ The Fourier spectrum of coherences (FSC), obtained via Fourier transform of the residuals after integrating the 2D maps along both frequency dimensions,^48^ reveals that the oscillations in the 2D signal of SQ are dominated by beatings at ν_1_ = 166 cm^–1^, ν_2_ = 574 cm^–1^ and ν_3_ = 1353 cm^–1^, as shown in Figure 4a. A fourth mode above the noise level could be detected at about 950 cm^–1^. This mode is due to a nonresonant contribution of the acetonitrile solvent and will not be considered further in the following analysis. The same beating components were also captured by the global fitting analysis (blue fitting curve in Figure 2b–d), supporting the reliability of the obtained results. This analysis also allows to obtain information on the dephasing time and amplitude distribution of these beating components across the 2D maps. All three relevant beating components exhibit relatively long dephasing times, on the order of several hundred femtoseconds, suggesting their origin lies in vibrational modes of the molecule, as expected for an organic chromophore in solution. This assignment is further supported by the close correspondence of these frequencies with both experimental and computed Raman spectra.^9,49^ These vibrational modes were assigned based ab initio vibrational analysis as follows (normal mode visualizations are provided in Figure 5a): ν_1_ corresponds to a collective rocking mode of the *N*,*N*′-diisobutyl methylenic groups directly bonded to the nitrogen atom (computed at 159.04 cm^–1^); ν_2_ involves a breathing motion of phenolic rings combined with a distortion of the squaric core (computed at 579.79 cm^–1^); and ν_3_ represents the symmetric O–H bending modes in the intermolecular hydrogen bond network (computed at 1360.84 cm^–1^).

**Figure 4:**
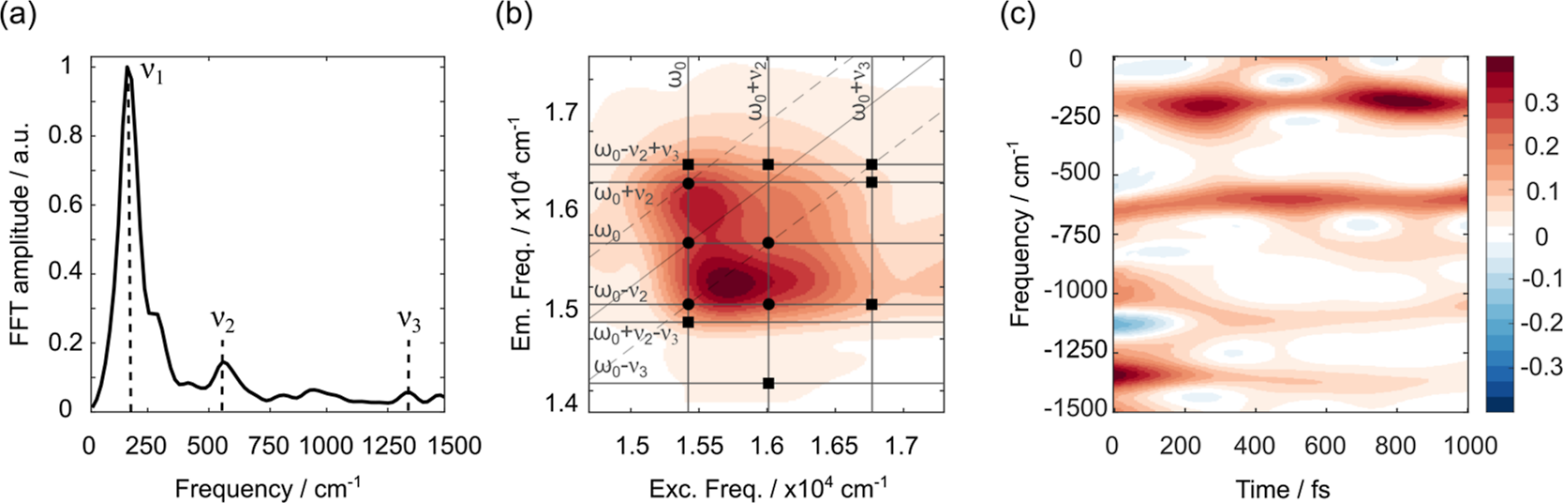
(a) Fourier spectrum of coherences, retrieved from the analysis of the oscillating residuals, averaged over all coordinates of the 2D maps; the amplitude is normalized on the most intense ν_1_ peak. The main oscillating frequencies (ν_1_ = 166 cm^–1^, ν_2_ = 574 cm^–1^ and ν_3_ = 1353 cm^–1^) are emphasized. (b) 2D-CAS of the ν_2_ mode (rephasing signal); black dots pinpoint the chair-pattern proper of ν_2_ while black squares account for the signal arising from the multimode coupling between ν_2_ and ν_3_ modes; ω_0_ = 15,420 cm^–1^. (c) Time-frequency transform analysis of the oscillating residuals extracted at coordinates (16,650, 14,600) cm^–1^. Additional 2D-CAS maps and TFT are reported in Supporting Information, Section S3.

**Figure 5:**
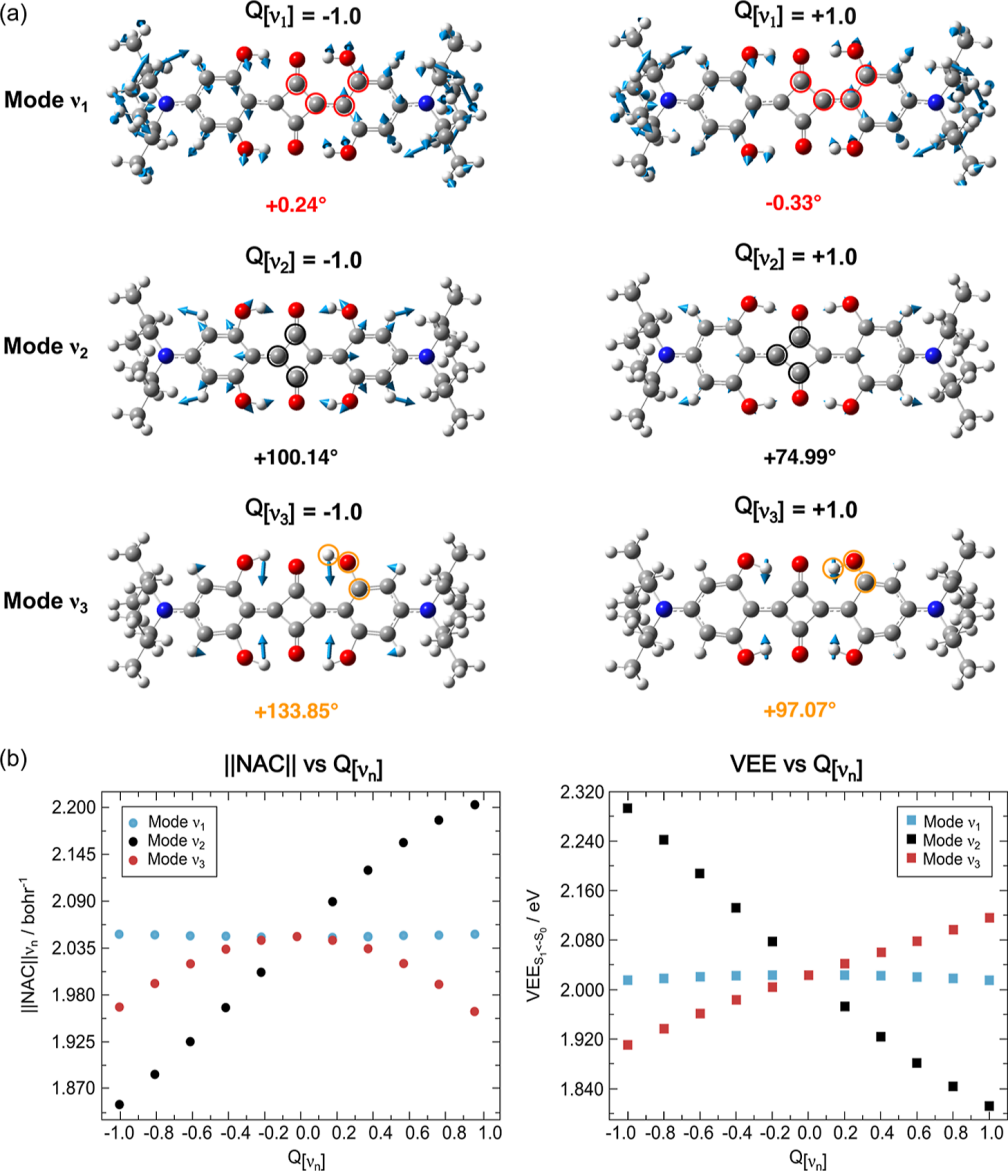
(a) Visualization of the main normal modes contributing to the beating behavior in SQ dynamics: ν_1_ = 166 cm^–1^, ν_2_ = 574 cm^–1^, and ν_3_ = 1353 cm^–1^. For each mode, the distorted structures at $Q_{\left[\nu_{n}\right]}$ = −1.00 (left column) and $Q_{\left[\nu_{n}\right]}$ = +1.00 (right column) are shown, where $Q_{\left[\nu_{n}\right]}$ is the normal mode displacement coordinate for the n-th vibrational mode. Corresponding values of selected geometrical parameters are provided, including dihedral angles (pinpointed by red circles) and bond angles (black and orange circles). Atom color code: carbon—dark gray; hydrogen—white; nitrogen—blue; oxygen—red. (b) LR-TDDFT analysis: first-order nonadiabatic coupling vector, ∥NAC∥ (left panel, shown as magnitude with rounded dots) and vertical excitation energy, VEE (right panel, square dots), along the displacements of ν_1_ (light blue), ν_2_ (black), and ν_3_ (red) modes. See Section S4 of the Supporting Information for further details on the theoretical level of calculation. ∥NAC∥ is reported in bohr^–1^, and VEE in eV.

Plotting the amplitude distribution associated with each vibrational mode yielded the coherence-associated spectra (2D-CAS),^12^ which are fully reported in the Supporting Information (Figures S6 and S7). For all beating modes, the amplitude distribution in the 2D maps predominantly follows the characteristic “chair-like” pattern predicted by the displaced harmonic oscillator model for vibrational modes in molecules.^50^ However, a closer inspection reveals additional, distinctive features, as reported in Figure 4b. These extra signals appear at coordinates corresponding approximately to the sum and difference of the frequencies of two modes, suggesting the presence of combination signatures resulting from multimode coupling. The appearance of overtones and combination bands arising from vibrational coherences between multiple fundamental modes is well-documented in vibrational spectroscopy.^51–53^ Similar interactions between coupled vibrations have been shown to generate combination frequencies and new cross-peaks in other nonlinear spectroscopies as well.^54–56^ Interactions between nuclear modes have also been observed in 2DES measurements by numerous groups.^57–61^ While a more comprehensive analysis will require targeted future investigations beyond the scope of this work, the present experimental results provide strong evidence for a significant degree of anharmonicity—consistent with expectations in the presence of a CI.

As introduced before, the 2S2M model for CIs classifies the vibrational degrees of freedom involved in the formation of a CI into two categories: tuning and coupling modes. The propagation of nuclear wavepackets along these modes is predicted to be strongly and differently influenced by the presence of a CI.^62–64^ Specifically, the wavepacket dynamics along the coupling mode are expected to be significantly disrupted due to destructive interference occurring before and after the passage through the CI, as well as due to the pronounced anharmonicity in the vicinity of the intersection.^62^ These effects cause a rapid decay of vibrational coherences associated with the coupling mode, on the same time scale as the nonadiabatic transition through the CI. In contrast, coherences involving the tuning mode are not expected to be similarly affected by these interference effects.^64^

This theoretical framework implies that time-resolved monitoring of vibrational coherence dynamics could provide valuable insight into the presence and topology of the CI, allowing for experimental discrimination between tuning and coupling mode behavior.

To explore the time evolution of vibrational coherences, a time-frequency transform (TFT) analysis^65,66^ was performed. This approach overcomes the limitations of conventional Fourier transform-based methods by preserving both temporal and spectral resolution. In a TFT spectrum, the ordinate represents the frequencies of the components contributing to the beating pattern at a specific coordinate of the 2D map, analogous to a conventional Fourier spectrum, while the abscissa displays their time evolution.^65–67^

Figure 4c summarizes the results obtained by applying the TFT analysis to the decay trace extracted at relevant coordinates where contributions from all of the coupled vibrations are captured. The ν_2_ component at 574 cm^–1^ exhibits a relatively stable time evolution, as its intensity remains unaffected for all the investigated time window and shows no significant variations in the time scale expected for the crossing of the CI (167 fs). In contrast, the ν_3_ mode (1353 cm^–1^) is highly active during the first 200 fs, after which it undergoes significant damping. Based on these properties, it can be reasonably inferred that the ν_2_ mode, unaffected by the CI crossing, serves as a tuning mode, whereas the strong damping of the ν_3_ coherence within the time scale of the CI interaction identifies it as a coupling mode.^64^ More complex is the interpretation of the ν_1_ mode (166 cm^–1^) that displays a peculiar evolution with a beating intensity pattern with an approximate period of 600 fs. This behavior can be attributed to interference with another mode having a similar frequency, most likely the one originating the shoulder detected at 240 cm^–1^ in the FSC (Figure 4a). Its evolution does not appear to be influenced by the passage through the CI but it is interesting to underline how the vibrational period of ν_1_ (ca. 200 fs) closely matches the CI time constant, complicating the distinction between vibrational coherences formed before and after the CI crossing.^68^ Overall, this mode is characterized by a very strong coherent activity in the 2DES spectra, as shown in the FSC of Figure 4a. Previous works investigating the role of different vibrational modes during the CI crossing have proposed the existence of another class of modes associated with the CI. These are vibrational modes that are impulsively generated in the excited-state potential energy surface S_1_ (Figure 3) and correspond to nuclear motions orthogonal to the CI seam. Such vibrational coherences are not significantly affected by the crossing and can be coherently transferred into the lower electronic state (S_0_^′^), where they persist as so-called “spectator” modes. This mechanism has been observed in a variety of ultrafast nonradiative processes across different molecular systems.^43,69–71^ Furthermore, if the CI has a sloped topography, as proposed for SQ, the rapid passage through the intersection can place the system on a steep region of the S_0_^′^ potential energy surface, leading to enhanced vibrational coherence activity in these spectator modes,^64^ as indeed observed experimentally.

To support this interpretation, first-order nonadiabatic coupling (NAC) vectors as well as vertical excitation energies (VEE) for the electronic transition responsible of the absorption (S_1_ ← S_0_)^9^ were computed by exploiting the liner-response time-dependent (LR-TD) DFT framework.^9,22,72,73^ Such quantities are reported and analyzed for several structures starting from the minimum energy one and distorted along the ground state normal modes (for further details about calculations, see Section S4 in the Supporting Information), corresponding to the ν_*n*_ (*n* = 1, 2, 3).

The obtained results, summarized in Figure 5b, show that for the ν_3_ mode, the NAC (red rounded dots) exhibits a parabolic dependence on the mode coordinate, while VEE (red squares) varies linearly. These trends suggest that the mode-induced distortion enhances the wave function overlap between the two electronic states, thereby modulating the coupling between S_0_ and S_1_ and supporting the assignment of ν_3_ as a coupling mode.

As shown in Figure 5a, this mode primarily involves bending motions of the O–H groups forming the intermolecular hydrogen-bond network. It is therefore plausible that displacement along this mode contributes to the breaking of the hydrogen-bond network along the excited-state potential energy surface—a type of structural evolution consistent with the distorted geometry we associate with a sloped intersection topography, as previously discussed. In contrast, for the ν_2_ mode, a significant reduction of the S_0_–S_1_ energy gap (black squares) along with the simultaneous linear increment of the corresponding NAC (black rounded dots) are observed along the vibrational distortion. This behavior indicates that ν_2_ contributes to the overall reaction coordinate through a pronounced distortion of the squaric ring, consistent with its identification as a tuning mode. Finally, for the ν_1_, no substantial variation in NAC and VEE (light blue rounded dots and squares, respectively) is detected along its coordinate, reinforcing the conclusion that most likely it does not participate directly in the CI branching dynamics.

## Conclusions

This work investigated the ultrafast excited-state dynamics of a prototypical red-absorbing squaraine dye, using 2DES in conjunction with ab initio simulations. Our findings provide compelling experimental evidence for the involvement of a CI in the nonradiative decay pathway of SQ, a feature previously hypothesized but not directly observed in this class of dyes. Through global analysis and time-frequency transform techniques, we identified vibrational coherences and their time evolution. Comparison with simulation results enabled the assignment of specific vibrational modes to the tuning and coupling coordinates of the CI branching space. Our analysis suggests that CI-mediated relaxation accounts for approximately 20% of the excited-state decay, indicating that while the CI plays a significant role, it does not dominate the photophysics of SQ. This partial accessibility is attributed to structural rigidity and intramolecular hydrogen bonding that stabilize the emissive S_1_ state, enabling both fluorescence and nonradiative decay pathways to coexist. From a practical perspective, these findings have important implications for the design of squaraine-based dyes in DSSCs and underscore the importance of considering conical intersections as tunable photophysical elements in dye design. Overall, this study showcases the power of 2DES combined with quantum chemical modeling in elucidating complex excited-state processes and provides a roadmap for exploring nonadiabatic dynamics in other organic photoactive systems.

## Supplementary Material



## Notes

Additional experimental details on 2DES setup; additional 2DES data; time-resolved fluorescence characterization of SQ in acetonitrile; computational details (PDF)
