# Supplementary material for: Signatures of a Conical Intersection in Two-Dimensional Spectra of a Red-Absorbing Squaraine Dye
Source: J Am Chem Soc. 2025 Aug 26;147(36):32995–3003. doi: 10.1021/jacs.5c10393 (PMC12426925; doi:10.1021/jacs.5c10393)
Supplement: Supplementary file 1 [file ja5c10393_si_001.pdf]

SUPPLEMENTARY INFORMATION
Signatures of a Conical Intersection in Two-dimensional Spectra of a Red-absorbing Squaraine Dye

Vittoria Burigana,[†] Edoardo Buttarazzi,[‡,¶] Federico Toffoletti,[†] Elisa Fresch,[†] Francesco Tumbarello,[†] Alessio Petrone,[¶,‡,§] and Elisabetta Collini[*,†]

†*Department of Chemical Sciences, University of Padova, Via Marzolo 1, I-35131 Padova, Italy*

‡*Scuola Superiore Meridionale, Largo San Marcellino 10, I-80138 Napoli, Italy*

¶*Department of Chemical Sciences, University of Napoli Federico II, Complesso Universitario di Monte S. Angelo, Via Cintia 21, I-80126 Napoli, Italy*

§*Istituto Nazionale Di Fisica Nucleare, Sezione di Napoli, Complesso Universitario di Monte S. Angelo ed. 6, Via Cintia, I-80126 Napoli, Italy*

E-mail: elisabetta.collini@unipd.it

S1. Time-resolved fluorescence

The lifetime of SQ in acetonitrile was measured via time-correlated single photon counting (TCSPC) technique. The data were collected with a modified version of a HORIBA® FluoroMax, equipped with a pulsed nanoLED centered at 609 nm with a 1 MHz repetition rate and a 1.5 ns time resolution. The measured lifetime is (2.5 ± 0.2) ns. The corresponding

data and fitting are reported in Figure S1.

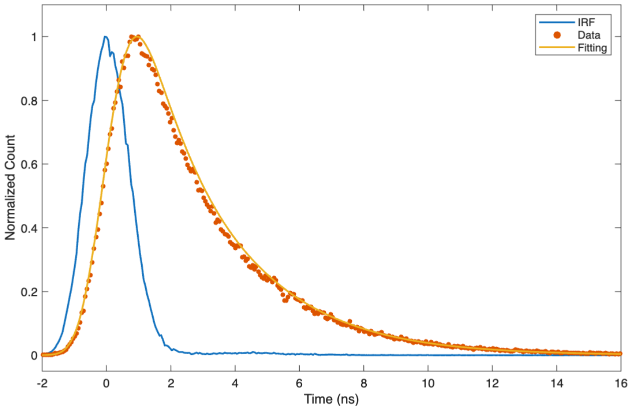

Figure S1: TCSPC data of SQ in acetonitrile. Orange dots: experimental data; blue solid line: instrumental response function (IRF); yellow solid line: mono-exponential fitting curve.

S2. 2DES Setup

The 2DES experiments were performed using a 3 kHz Ti:Sapphire Coherent® Libra laser system, coupled with a commercial NOPA (Light Conversion® TOPAS White). A pulse compression of approximately 10 fs at the sample position was achieved using a prism compressor in combination with a Fastlite Dazzler pulse shaper for fine temporal tuning. The final pulse duration was optimized using Transient Grating Frequency-Resolved Optical Gating (TG-FROG) measurements performed on dimethyl sulfoxide (DMSO) solvent (see Figure S2). The retrieved pulse trace was analyzed using a custom MATLAB script to extract the temporal profile and confirm compression quality.[1] The pulse energy at the sample position was attenuated to ∼7 nJ per pulse using a broadband half-wave plate and polarizer assembly. The 2DES setup employed a passively phase-stabilized configuration in a BOXCARS geometry. The laser output was split into four phase-stable beams: three for sample excitation and one used as the local oscillator (LO) for heterodyne detection. Beam splitting and spatial alignment were facilitated by a custom-designed two-dimensional grating. Temporal delays between pulses were controlled by pairs of CaF_2 wedges, with one wedge in each pair mounted on a translation stage, allowing fine adjustment of optical path length.

This enabled sub-femtosecond delay precision (temporal resolution $\sim$0.07 fs). The coherence time (t_1, delay between the first and second pulses), population time (t_2, delay between the second and third pulses), and rephasing time (t_3, delay between the third pulse and the signal emission) were defined for each measurement. Both rephasing and non-rephasing signal components were recorded by altering the pulse sequence. Their combination enabled the reconstruction of purely absorptive 2D maps. The data acquired comprise a 3D array describing the evolution of 2D frequency–frequency correlation spectra as a function of t_2. In each map, the excitation (ω_{exc}) and detection (ω_{det}) frequency axes result from Fourier transformation along t_1 and t_3, respectively. Fixed slices along t_2 yield the 2D maps that reveal the dynamic evolution of electronic states and couplings. The population time t_2 was scanned from 0 to 1000 fs in 7.5 fs increments. The coherence time t_1 was scanned from 0 to 90 fs (step size: 2.3 fs). A rotating frame approach was applied. Each experiment was repeated at least five times, and the results were averaged to ensure reproducibility and reduce noise. For a detailed description of the experimental setup, see Ref.[1]

The experiments were performed at room temperature, employing an excitation laser with a bandwidth centered at 15870 cm^{-1} (630 nm), approximately 100 nm in width. The optical density of the sample was 0.12 with an optical path length of 1 mm.

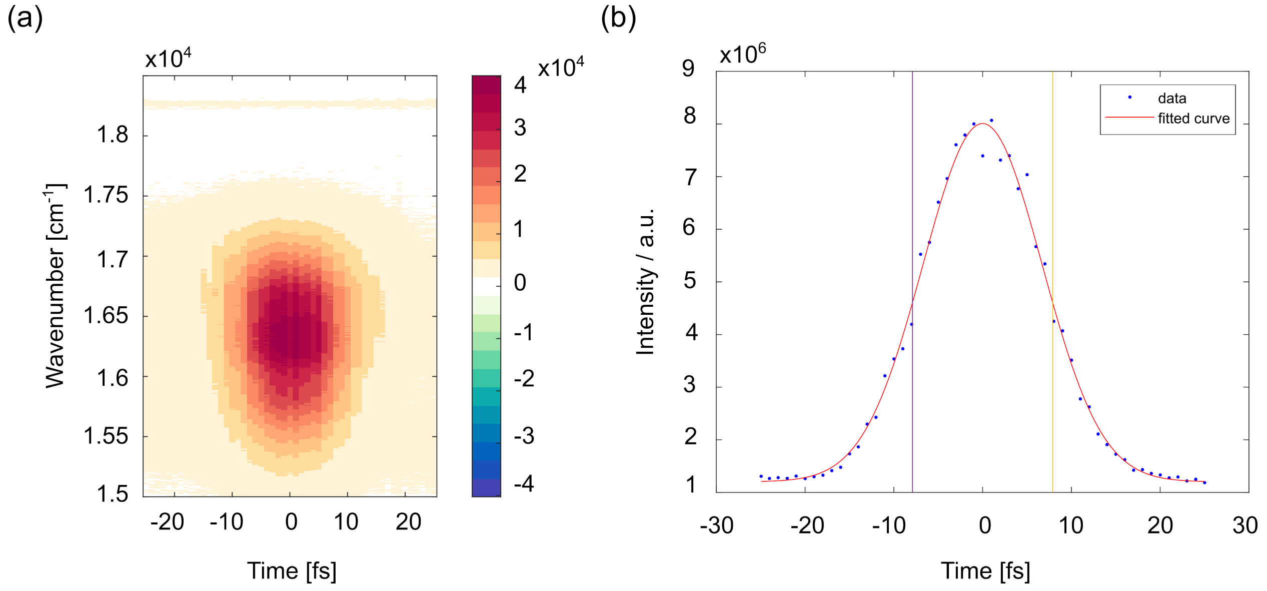

Figure S2: Pulse characterization by TG-FROG measurements. (a) Experimental pulse profile measured by the FROG experiment showing no relevant chirp effects. (b) Fitting of the FROG signal (integrated along the frequency axis) with a Gaussian function.

S3. Additional 2DES data

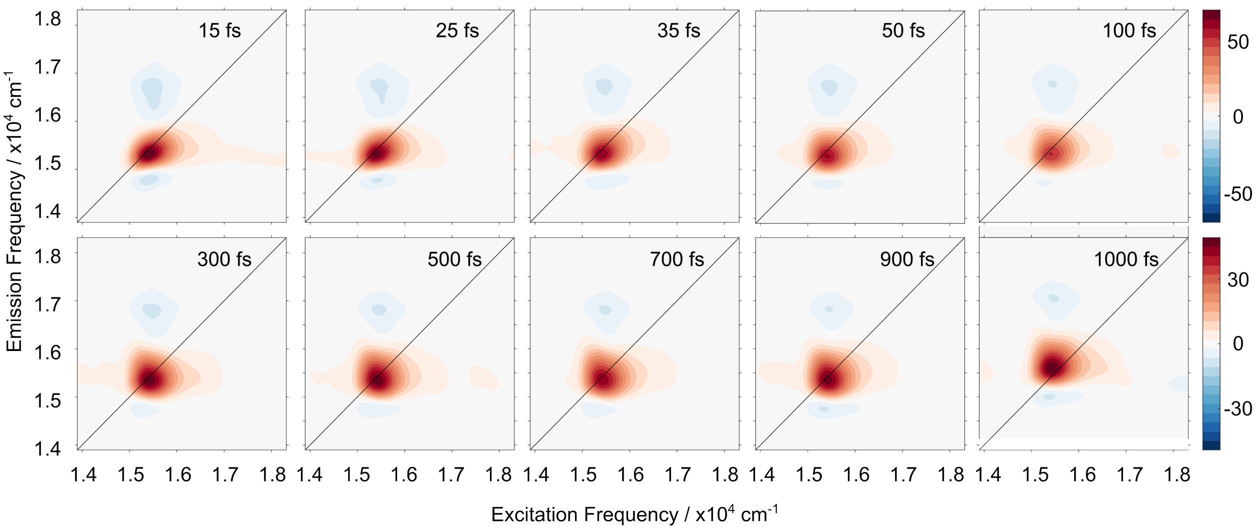

Figure S3: Purely absorptive 2D maps for SQ in acetonitrile recorded for the population times indicated in each panel.

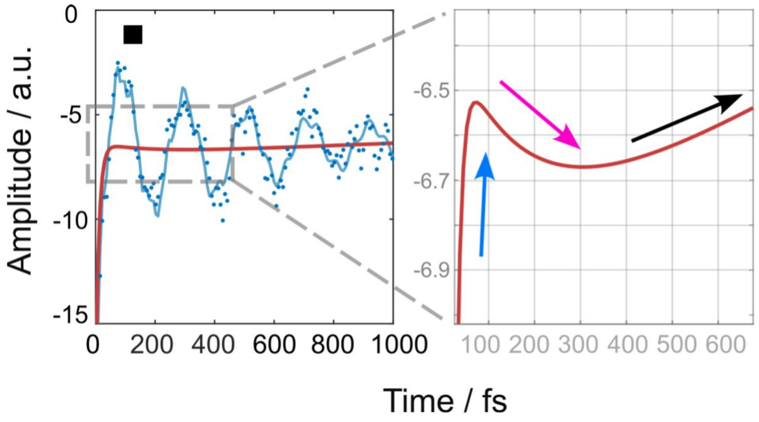

Figure S4: (Left) Signal decay trace extracted at coordinates (15420, 14760) cm^{-1} (same as in Figure 2d). Dotted line: experimental data; blue solid line: global fit including both vibrational beatings and decay components; red solid line: fit including only decay components. (Right) Zoomed-in view of the fitting curve in the 0–600 fs time interval, highlighting the presence of three distinct dynamical processes, indicated by colored arrows. Blue arrow: within the first ∼20 fs, the signal rapidly recovers about half of its amplitude; pink arrow: between ∼20 and 400 fs, the signal becomes more negative; black arrow: from ∼400 fs onward, the signal decays—i.e., the amplitude gradually becomes more positive and approaches zero.

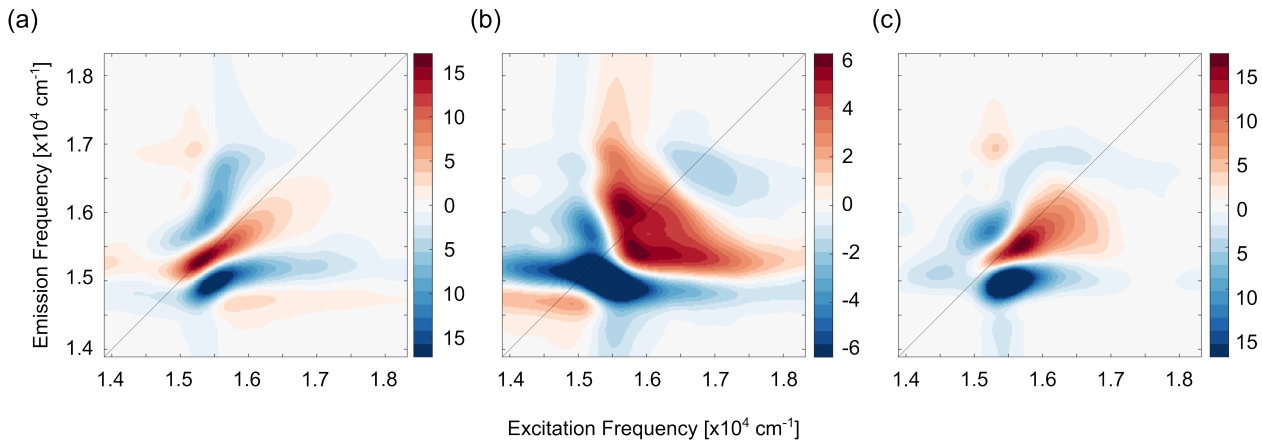

Figure S5: 2D-DAS corresponding to the 15 fs time constant for the (a) Rephasing, (b) Non-Rephasing, and (c) Total signals.

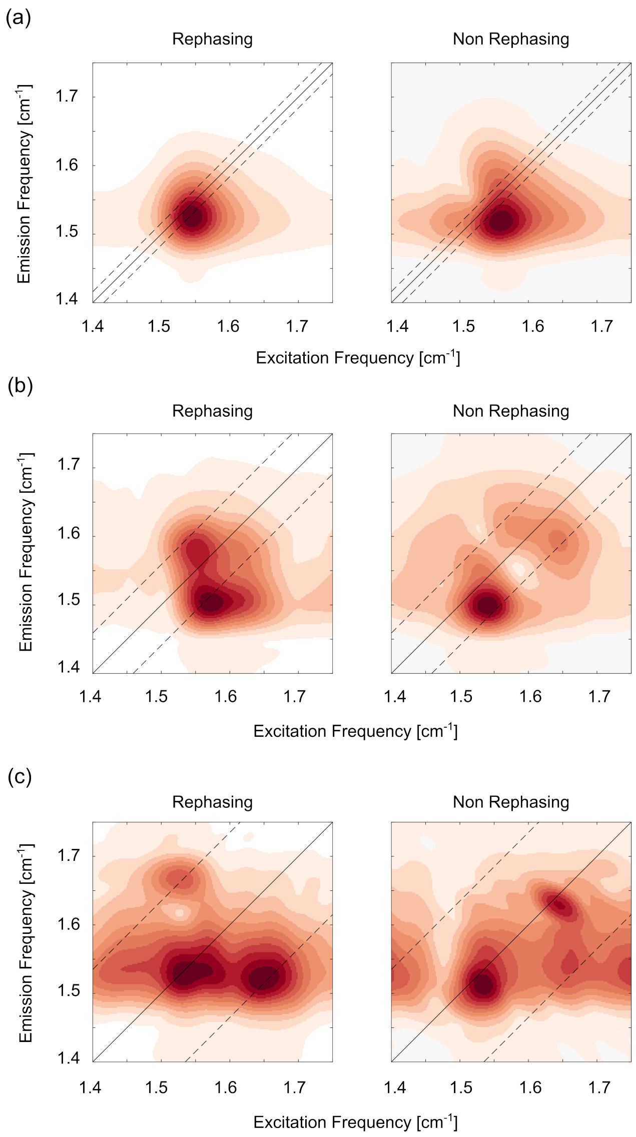

Figure S6: Normalized 2D-CAS (absolute values) relative to the three main vibrational modes coupled with the electronic transition for SQ in acetonitrile: (a) ν_1=166 cm^{-1}; (b) ν_2=574 cm^{-1}; (c) ν_3=1353 cm^{-1}. In each panel, positive and negative frequencies are summed.

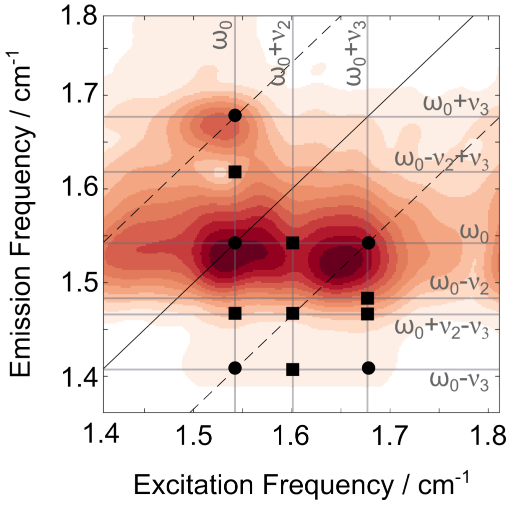

Figure S7: Normalized rephasing 2D-CAS spectrum (positive and negative frequencies summed) for mode ν_3. Black dots mark the characteristic "chair" pattern at the ν_3 frequency, as predicted by the displaced harmonic oscillator model. Black squares indicate positions where the signal arises from multi-mode coupling with ν_2. Gray lines highlight the relevant frequency combination coordinates (ω_0=15420 cm^{-1}).

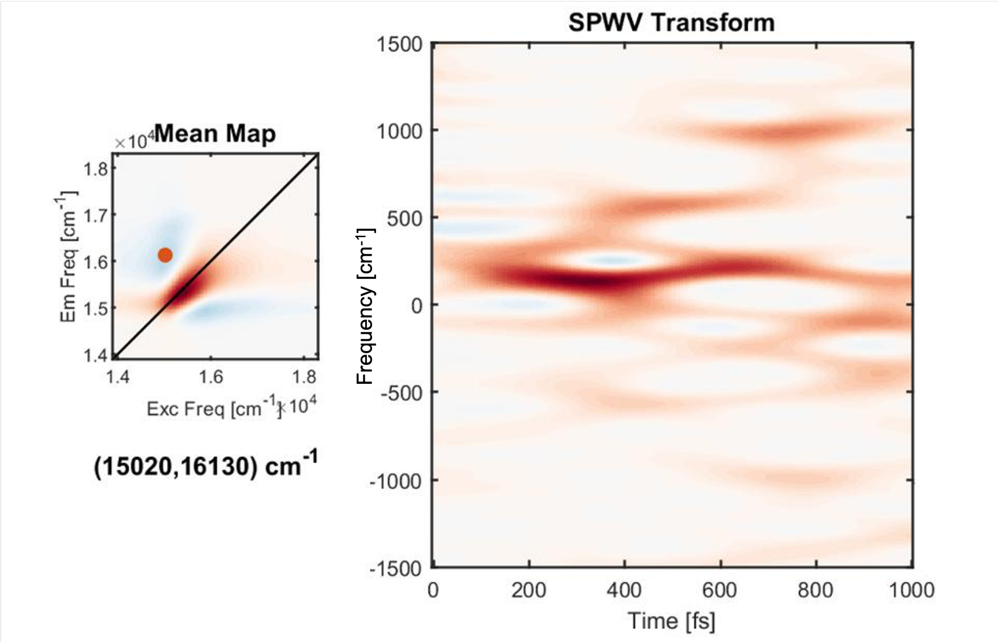

Figure S8: Time-frequency transform analysis of the oscillating residuals extracted at coordinates (15020, 16130) cm^{-1}

.

S4. Computational details

2,4–Bis[4–(N,N–diisobutylamino)–2,6–dihydroxyphenyl]squaraine (SQ) was treated at DFT level of theory and all calculations were performed using the Gaussian 16 electronic structure software package, version C.01.[2] Electronic structure calculations were carried out by solving the Kohn–Sham equations using the global hybrid Becke 3–parameter Lee–Yang–Parr, B3LYP,[3–5] for the minimum energy structure and the harmonic vibrational analysis. Excited state properties were computed through the linear response-time dependent DFT (LR–TDDFT) formalism,[6–8] employing the long–range corrected version of B3LYP using the Coulomb–attenuating method, CAM–B3LYP.[9] In combination with both levels of theory, the Pople's 6–31+G(d,p) basis set was used.[10–13] This combination of levels of theory has been validated for the characterization of vibrational and optical properties in solution for the selected molecular system.[14] Acetonitrile solvent effects were included using implicit solvation models, particularly the conductor–like polarizable continuum model (C–PCM).[15–19] Geometry was considered fully optimized when both the force (maximum and RMS force, 0.000450 and 0.000300 Hartree bohr^{-1} thresholds, respectively) and displacement (maximum and RMS displacement, 0.0018 and 0.0012 bohr thresholds, respectively) values for all atoms were below the threshold criteria. The B3LYP minimum energy structure is available in the Supplementary Materials of Ref.,[14] and was checked to be a true minimum, by computing geometrical Hessian and diagonalizing it, checking that the resulting vibrational frequencies were all positive. First–order Cartesian nonadiabatic coupling matrix elements (NACs)[20,21] are defined by:

$$d^i_{01} = \langle \Phi_0 | \frac{\partial}{\partial x_i} | \Phi_1 \rangle$$

where x_i is the i–th atomic cartesian nuclear coordinate, and Φ_0, Φ_1 are the ground and first excited electronic states, respectively.

NACs were computed analytically using the LR–TDDFT formalism as described in

Refs.[21–24] To identify which internal molecular vibrations contribute to nonradiative decay and facilitate approach to the conical intersection, we selected a few normal modes from the vibrational analysis (ν_1,ν_2 and, ν_3) and computed the corresponding NAC matrix elements and vertical excitation energies along these distortions.

In detail, NAC analysis was carried out on several structures displaced by several fundamental harmonic vibrational modes (i.e. $Q_{[\nu_n]}$, pure scalar number). $Q_{[\nu_n]}$ is the normal displacement value for the n–th vibrational mode, the range value is the same for all selected modes: ± 1.00 with displacement resolution of 0.20.

The Results and Discussion section reports the Frobenius norm of the NAC matrix ($\|$NAC$\|$) and vertical excitation energy (VEE, from the ground to the first singlet excited state) scans at each displaced geometry along the corresponding vibrational mode.

References

(1) Bolzonello, L.; Volpato, A.; Meneghin, E.; Collini, E. Versatile setup for high-quality rephasing, non-rephasing, and double quantum 2D electronic spectroscopy. *JOSA B* **2017**, *34*, 1223–1233.

(2) Frisch, M. J.; Trucks, G. W.; Schlegel, H. B.; Scuseria, G. E.; Robb, M. A.; Cheeseman, J. R.; Scalmani, G.; Barone, V.; Petersson, G. A.; others Gaussian 16 Revision C.01. 2016; Gaussian Inc. Wallingford CT.

(3) Lee, C.; Yang, W.; Parr, R. G. Development of the Colle-Salvetti correlation-energy formula into a functional of the electron density. *Physical Review B* **1988**, *37*, 785–789.

(4) Miehlich, B.; Savin, A.; Stoll, H.; Preuss, H. Results obtained with the correlation energy density functionals of Becke and Lee, Yang and Parr. *Chemical Physics Letters* **1989**, *157*, 200–206.

(5) Becke, A. D. Density-functional thermochemistry. III. The role of exact exchange. *The Journal of Chemical Physics* **1993**, *98*, 5648–5652.

(6) Casida, M. E. *Recent Advances in Density Functional Methods*; World Scientific Publishing, 1995; pp 155–192.

(7) Stratmann, R. E.; Scuseria, G. E.; Frisch, M. J. An efficient implementation of time-dependent density-functional theory for the calculation of excitation energies of large molecules. *The Journal of Chemical Physics* **1998**, *109*, 8218–8224.

(8) Dreuw, A.; Head-Gordon, M. Single-reference ab initio methods for the calculation of excited states of large molecules. *Chemical Reviews* **2005**, *105*, 4009–4037.

(9) Yanai, T.; Tew, D. P.; Handy, N. C. A new hybrid exchange-correlation functional using the Coulomb-attenuating method (CAM-B3LYP). *Chemical Physics Letters* **2004**, *393*, 51–57.

(10) Ditchfield, R.; Hehre, W. J.; Pople, J. A. Self-consistent molecular-orbital methods. IX. An extended Gaussian-type basis for molecular-orbital studies of organic molecules. *J. Chem. Phys.* **1971**, *54*, 724–728.

(11) Hehre, W. J.; Ditchfield, R.; Pople, J. A. Self-consistent molecular orbital methods. XII. Further extensions of Gaussian-type basis sets for use in molecular orbital studies of organic molecules. *The Journal of Chemical Physics* **1972**, *56*, 2257–2261.

(12) Hariharan, P. C.; Pople, J. A. The influence of polarization functions on molecular orbital hydrogenation energies. *Theoretica chimica acta* **1973**, *28*, 213–222.

(13) Clark, T.; Chandrasekhar, J.; Spitznagel, G. W.; Schleyer, P. V. R. Efficient diffuse function-augmented basis sets for anion calculations. III. The 3-21+G basis set for first-row elements, Li–F. *Journal of Computational Chemistry* **1983**, *4*, 294–301.

(14) Buttarazzi, E.; Inchingolo, A.; Pedron, D.; Alberto, M. E.; Collini, E.; Petrone, A. Conformational and environmental effects on the electronic and vibrational properties of dyes for solar cell devices. *The Journal of Chemical Physics* **2024**, *160*, 204301.

(15) Mennucci, B.; Tomasi, J. Continuum solvation models: A new approach to the problem of solute's charge distribution and cavity boundaries. *The Journal of Chemical Physics* **1997**, *106*, 5151–5158.

(16) Barone, V.; Cossi, M. Quantum calculation of molecular energies and energy gradients in solution by a conductor solvent model. *The Journal of Physical Chemistry A* **1998**, *102*, 1995–2001.

(17) Cossi, M.; Scalmani, G.; Rega, N.; Barone, V. New developments in the polarizable continuum model for quantum mechanical and classical calculations on molecules in solution. *The Journal of Chemical Physics* **2002**, *117*, 43–54.

(18) Cossi, M.; Rega, N.; Scalmani, G.; Barone, V. Energies, structures, and electronic properties of molecules in solution with the C-PCM solvation model. *Journal of Computational Chemistry* **2003**, *24*, 669–681.

(19) Cossi, M.; Rega, N.; Scalmani, G.; Barone, V. Polarizable dielectric model of solvation with inclusion of charge penetration effects. *The Journal of Chemical Physics* **2001**, *114*, 5691–5701.

(20) Send, R.; Furche, F. First-order nonadiabatic couplings from time-dependent hybrid density functional response theory: Consistent formalism, implementation, and performance. *The Journal of Chemical Physics* **2010**, *132*, 044107.

(21) Chernyak, V.; Mukamel, S. Density-matrix representation of nonadiabatic couplings in time-dependent density functional (TDDFT) theories. *The Journal of Chemical Physics* **2000**, *112*, 3572–3579.

(22) Li, Z.; Suo, B.; Liu, W. First order nonadiabatic coupling matrix elements between excited states: Implementation and application at the TD-DFT and Pp-TDA levels. *The Journal of Chemical Physics* **2014**, *141*, 244105.

(23) Wang, Z.; Wu, C.; Liu, W. NAC-TDDFT: Time-dependent density functional theory for nonadiabatic couplings. *Accounts of Chemical Research* **2021**, *54*, 3288–3297.

(24) Coppola, F.; Cimino, P.; Raucci, U.; Chiariello, M. G.; Petrone, A.; Rega, N. Exploring the Franck–Condon region of a photoexcited charge transfer complex in solution to interpret femtosecond stimulated Raman spectroscopy: Excited state electronic structure methods to unveil non-radiative pathways. *Chemical Science* **2021**, *12*, 8058–8072.
